# A New Method to Predict the Epidemiology of Fungal Keratitis by Monitoring the Sales Distribution of Antifungal Eye Drops in Brazil

**DOI:** 10.1371/journal.pone.0033775

**Published:** 2012-03-23

**Authors:** Marlon Moraes Ibrahim, Rafael de Angelis, Acacio Souza Lima, Glauco Dreyer Viana de Carvalho, Fuad Moraes Ibrahim, Leonardo Tannus Malki, Marina de Paula Bichuete, Wellington de Paula Martins, Eduardo Melani Rocha

**Affiliations:** 1 Department of Ophthalmology, Faculty of Medicine of Ribeirão Preto, University of São Paulo, Ribeirão Preto, Brazil; 2 Department of Ophthalmology, São Paulo Federal University, São Paulo, Brazil; 3 Ophthalmos Industria Farmacêutica, São Paulo, Brazil; 4 Departamento de Ginecologia e Obstetrícia da Faculdade de Medicina de Ribeirão Preto, Universidade de São Paulo, Ribeirão Preto, Brazil; 5 Escola de Ultra-sonografia e Reciclagem Médica de Ribeirão Preto, Ribeirão Preto, Brazil; 6 Instituto Nacional de Ciência e Tecnologia de Hormônios e Saúde da Mulher, Ribeirão Preto, Brazil; Kenya Medical Research Institute - Wellcome Trust Research Programme, Kenya

## Abstract

**Purpose:**

Fungi are a major cause of keratitis, although few medications are licensed for their treatment. The aim of this study is to observe the variation in commercialisation of antifungal eye drops, and to predict the seasonal distribution of fungal keratitis in Brazil.

**Methods:**

Data from a retrospective study of antifungal eye drops sales from the only pharmaceutical ophthalmologic laboratory, authorized to dispense them in Brazil (Opthalmos) were gathered. These data were correlated with geographic and seasonal distribution of fungal keratitis in Brazil between July 2002 and June 2008.

**Results:**

A total of 26,087 antifungal eye drop units were sold, with a mean of 2.3 per patient. There was significant variation in antifungal sales during the year (*p<*0.01). A linear regression model displayed a significant association between reduced relative humidity and antifungal drug sales (R2 = 0.17,p<0.01).

**Conclusions:**

Antifungal eye drops sales suggest that there is a seasonal distribution of fungal keratitis. A possible interpretation is that the third quarter of the year (a period when the climate is drier), when agricultural activity is more intense in Brazil, suggests a correlation with a higher incidence of fungal keratitis. A similar model could be applied to other diseases, that are managed with unique, or few, and monitorable medications to predict epidemiological aspects.

## Introduction

Fungal keratitis is a common and important cause of corneal morbidity in tropical regions of the world. Perhaps because it is less common among industrialized countries located in more temperate climates, research into the risk factors and treatment options of this orphan disease has sometimes been lower than expected [1], [2].

In tropical and developing countries, fungal ulcers represent from 4% to 60% of infectious corneal ulcers [1], [3]–[5]. This distribution is believed to result from socioeconomic conditions, environmental characteristics, and geographical variations, such as latitude and climatic differences, especially humidity [1], [6]–[8]. Previous works has revealed a significant increase in the number of reported cases of fungal keratitis during the harvest period [1], [2], [8], [9].

Brazil is a developing tropical country with extensive dimensions and various environmental, climatic, and socio-economic characteristics along its length. According to the Köppen system [10], Brazil hosts five major climatic subtypes: equatorial, tropical, semiarid, highland tropical, and temperate. Biomes range from equatorial rainforests in the north and semiarid in the northeast, to temperate coniferous forests in the south and tropical savannas in central Brazil, but the largest part of the country is tropical.

*Fusarium* species of fungi are commonly associated with fungal keratitis in Brazil [3], [11], [12], [13], where natamycin and amphotericin B eye drops are available for the management of fungal keratitis. Indeed, these drops are widely prescribed [12]. Natamycin is the only topical ophthalmic antifungal agent approved by the US Food and Drug Administration, and is the drug of choice for filamentous fungal keratitis worldwide [14]. The commercialisation of antifungal eye drops is tightly regulated in Brazil; these drugs are not available in drugstores and must be exclusively formulated for each patient. Ophtalmos was the first and major laboratory authorised by ANVISA (National Health Surveillance Agency) to produce, market and distribute eye drops, that are not manufactured by the pharmaceutical industry. Therefore, the company provides most of the eye drops used by patients. The alternative is the manipulation (dilution) of a commercial presentation for systemic (intravenous) use by the physician or someone from his/her staff.

Kaiserman et al [15] previously used drug prescriptions to predict the prevalence of eye disease in diabetic patients, however, a similar approach to identify the geographical distribution and correlation with potential risk factors has not been used in ophthalmology.

The current study aims to examine the commercialisation of formulated antifungal eye drops, by the only pharmaceutical company formally able to distribute them in this country, and to predict the distribution and seasonality of fungal keratitis in Brazil.

## Methods

The Research Ethics Committee of the Faculty of Medicine of Ribeirão Preto, University of São Paulo approved this study, and it was conducted in accordance with the guidelines for confidentiality of medical records and the Declaration of Helsinki.

Antifungal eye drops are not commercially available in drugstores in Brazil. Rather, these drugs must be exclusively formulated for each patient and are sold directly by the laboratory, either through a call-in prescription or by postal delivery.

Antifungal eye drops are commercialised mainly by Ophtalmos Labs, in Brazil and dispensed only with the retention of prescription. We retrospectively analysed sales of natamycin and amphotericin B from this laboratory between July 2002 and June 2008. The company data bank of antifungal eye drops was analyzed and the distribution of dispensed units was registered by number of units/month for each drug and for each state of the federation. Population data were obtained from IBGE (Brazilian Institute of Geography and Statistics) at www.ibge.gov.br. The antifungal prescription was not necessarily made after microbiological confirmation of the aetiological agent and the clinical judgment to initiate or to continue antibiotic concomitant treatment was not evaluated.

We evaluated the data regarding units of antifungal eye drops sold during 72 months (6 years) for each one of the 27 Brazilian States. The total of units sold in each month was divided by the State population to reduce heterogeneity caused by different population size between States by using the following formula: antifungal eye drop units sold per million inhabitants  =  total units sold in one month/population of the State ×1,000,000. For each of the 72 months, data regarding total rain precipitation (mm), average relative humidity (%) and average temperature (°C) were also acquired for all 27 States. Climatic characteristics of each region were obtained from INMET (National Institute of Weather) at www.inmet.gov.br.

Statistical analysis was performed by using GraphPad Prism 5.0 (GraphPad Inc., San Diego, CA, USA) and SPSS 16.0 for Windows (SPSS Inc. Chicago, IL, USA). We evaluated the linear regression (R^2^ and p-value) between the antifungal eye drop sales per million inhabitants and total rain precipitation (mm), average relative humidity (%) and average temperature (°C) considering all 1,944 State-months. Additionally we performed multiple linear regression (stepwise model) between the same parameters.

When comparing antifungal eye drop sales differences between months, we performed one-way analysis of variance (ANOVA) considering the total units sold in the whole country for each month averaging for the 6-year period the outcome variable (dependent) and the months of the year the predictor (independent) variable. The Huber-White variance correction was applied in order to determine the robust estimator using each state as a subject and each of 72 months as repetition (12 months of 6 years). This analysis was performed using the Generalized Linear Models.

## Results

The antifungal eye drops sold were categorised into types: natamycin or amphotericin. There were more than five times as many sales of natamycin as there were sales of amphotericin throughout the study period and a total of 26,087 antifungal eye drop units were sold during the study period. In addition, the units per patient was almost double for natamycin compared to amphotericin (**Table 1**).

**Table 1:** Units of each antifungal eye drop sold between July 2002 and June 2008 in Brazil by Ophtalmos Labs.

Antifungal	Total Units	Total Patients	Units/Patient
Natamycin	21.707	7.688	2,82
Amphotericin B	4.380	2.487	1,76

Along of the 6 years evaluated there was no significant increase of antifungal eye drops sale.

The total number of individuals was 10175, dividing this number by the six years and the average Brazilian population in the period (188,298,099 inhabitants in 2006), it was estimated that the incidence of cases of fungal keratitis was 9.01/million of inhabitants per year [16].

**Figure 1:**
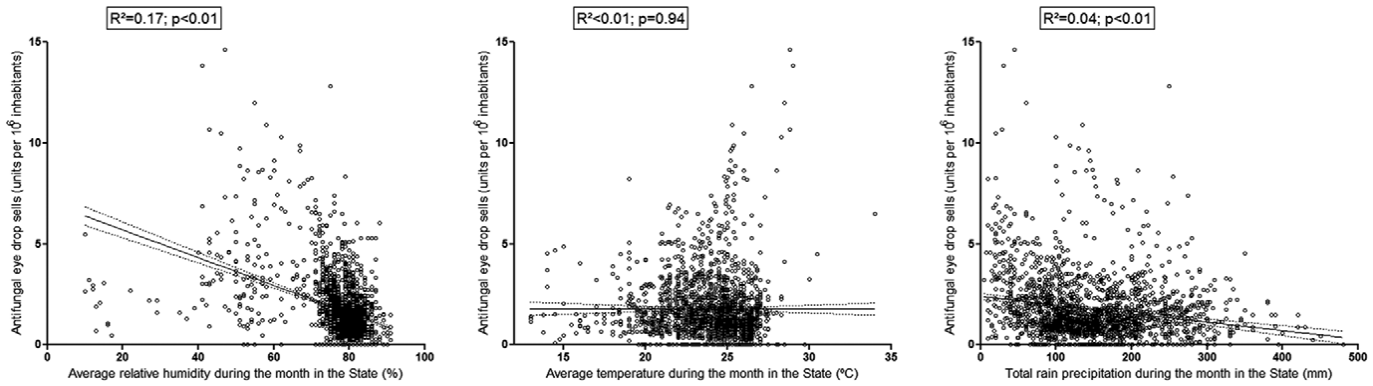
Plots of and the linear regression of the units of antifungal eye drop sold per million inhabitants with relative humidity, total rain precipitation and average temperature. The solid line represents the best fit curve and the dotted lines the 95% confidence interval.

When analysing the plots and the linear regression of the units of antifungal eye drop sold per million inhabitants with relative humidity, total rain precipitation and average temperature, we observed a significant association between the amount of antifungal eye drops units sold per inhabitant with both relative humidity and rain precipitation, but not with the temperature (**Figure 1**). When performing multiple linear regression, we observed that only relative humidity was associated with the amount of antifungal eye drops units sold per inhabitant (Beta = −0.41, p<0.01); after correction for humidity, there was no association of the amount of units sold per inhabitant and rain (Beta = −0.03, p = 0.17) or temperature (Beta = 0.04, p = 0.06).

**Figure 2:**
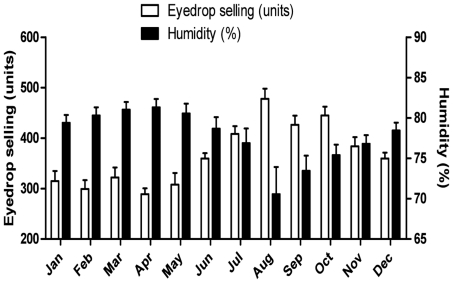
Antifungal eye drop units sold in Brazil (white columns) and the average relative humidity (black columns) separated by month averaged in 6-year period (from July 2002 to June 2008; white columns). Bars represent means and errors the 95% confidence interval.

We observed significant differences in the total of units of antifungal eye drop sold in Brazil between months during the 6-year period (p<0.01; ANOVA; **Figure 2**): the month with the highest sales was August (478±50 units; mean±SD); while the month with the lowest sales was April (289 ± 28 units; mean±SD). In **Figure 2** we also report the relative humidity in Brazil averaged in the 6-year period, demonstrating that the months with highest sales were also those with lowest relative humidity. Using Huber-White variance correction (robust estimator), there was significant correlation between sales and humidity (p<0.01), but not with temperature (p = 0.10) or rain precipitation (p = 0.23).

## Discussion

The estimated incidence of fungal keratitis n Brazil is much higher than reported in United Kingdom (UK) (9.01 versus 0.32 cases/million per year) [17]. However, the present study is probably overestimating the number of cases, since the survey here was based on number of individuals to whom the drugs were prescribed, and in UK, the numbers were related to microbiological confirmed cases. By now, it is not possible to make a similar survey, because there is not a national database for fungal keratitis and most of cases are treated based on clinical presentation. A recent study in Brazil estimated that 48% of clinically predicted fungal keratitis were confirmed by microbiological exam [18]. Even with those numbers, the incidence of fungal keratitis in Brazil would be more than 10 times higher than in UK. Moreover, the present work reveals that antifungal sales correlate with the driest season of the year.

Location and weather may play a major role in the epidemiology of eye diseases [19]. For instance, in more temperate climate areas, fungal corneal ulcers are more frequently caused by *Candida spp*. than by filamentous fungi [20]. However, this pattern is reversed in the tropics, where keratitis is predominantly caused by filamentous fungi. These geographic differences could be influenced by several factors, including climate, ecology, behavior, and socio-economics. In this study, we show that climate seems to play a substantial role for fungal keratitis, with humidity accounting for 17% of the variability in antifungal prescriptions in Brazil. [2], [12], [20], [21].

Studies have shown that areas where the climate is warm and humid, especially near the Equator, have more cases of fungal keratitis; in these areas, filamentous fungi are the dominant fungal corneal pathogen [12], [22]. Temperature and humidity have a major role in determining the microorganisms found in the environment; for example, *Fusaisum spp*. predominates in tropical areas [23], [24]. However, the specific seasonal behaviour was not previously documented. Observing that other important risk factors for fungal keratitis are agricultural activity and ocular vegetal injury and individuals have been shown to be more vulnerable to ocular trauma when harvesting or collecting, our findings agree with the hypothesis that in tropical region the driest season, when those activities are more frequent, present the highest frequency of antifungal eye drops sales [2].

Our study shows an increase in antifungal sales during the third trimester of the year in Brazil. This period coincides with the collection and harvest season for many crops, such as corn, soybeans, sugar cane, coffee, rice and oranges, however any correlation among our data and agricultural activity in Brazil is still speculative. Some researchers have shown that the incidence of fungal keratitis is higher during harvesting and also during other times of the year when agricultural activity is higher, thus correlating the disease with harvesting crops such as rice [25] and onion [7]. In Brazil, harvest time is a period when there is a low precipitation index, dry air and a lower mean temperature than at other times of the year. The peak incidence of fungal keratitis has already been correlated with windy and dry weather in other countries, where the principal fungus isolated from patients was *Fusarium* [2], [26]. However, studies in India have shown inverse disease seasonality, with disease incidence increasing during humid months [25], or even during the winter and monsoon (rainy) season [26], [27]. This discrepancy could be explained by a variety of factors. The causative organisms could be different in the 2 countries, with some Indian studies reporting high numbers of *Curvalaria* or *Aspergillus*, whereas Brazilian studies have reported mostly *Fusarium*. Though both are tropical countries, there are nonetheless ecological differences. For example, India has 6 major climatic subtypes, ranging from desert to glacial, whereas Brazil has only 5 major subtypes, and does not have either the desert or glacial subtype, according to the Köppen system [3], [8], [10], [27].

Although all five regions presented similar levels of antifungal topical eye drops sales and also comparable levels of humidity and rain precipitation, we saw high sales in Piaui, a state in the Northeast region, compared to the other states. It is one of the driest Brazilian states with a mean humidity of 70 ± 10.9%, while the national mean is 77.9 ± 7.4%. We did not see many sales of eye drops in the Amazon area, a place that is hot and wet, even though it is similar to places where a high incidence of fungal keratitis cases are seen. This low incidence may be explained by the low population density and predominant forest vegetation, both of which contribute to low levels of agricultural activity. In addition, a lower number of health care professionals in the region may explain the lower number of antifungal prescribed.

Combining these findings with the high incidence of filamentary fungal keratitis in males enrolled in agricultural work, it appears that fungal keratitis is a major labour–related disease [2].

Another relevant aspect in fungal keratitis is the paucity of therapeutical options and providers. In Brazil, this study was only possible because no industry commercializes antifungal eye drops and the only registered company dispenses them by prescriptions. It is possible that non-registered formulations, or patients treated with systemic drugs are a source of bias in the present work. However, the severity of fungal keratitis and the difficulty in resolving the infection generally leads patients to referral centers that comply with broadly accepted treatments.

Our results are consistent with other studies of fungal keratitis conducted in Brazil [12], [18] In previous studies, patients with fungal keratitis have on average been younger than patients with bacterial keratitis, which would be expected if younger agricultural workers are especially at risk for fungal keratitis. Moreover, previous studies have specifically identified ocular trauma and male gender as risk factors, which is consistent with the theory that agricultural work is the primary risk factor for fungal keratitis.

The observed trend in drug sales is a useful method to study seasonal distribution of filamentary fungal keratitis. The present data, combined with previous observations of a higher risk in individuals involved in agricultural work in Brazil, suggests relationship of this infection with agricultural routines, as well as the need to improve preventive methods, such as the use of protective glasses [12], [18]. This information should support the implementation of such strategies in vulnerable populations.
